# Evaluation of the association between mammographic density and the risk of breast cancer using Quantra software and the BI-RADS classification

**DOI:** 10.1097/MD.0000000000023112

**Published:** 2020-11-13

**Authors:** Jian Ming Wang, Hong Guang Zhao, Tong Tong Liu, Fei Yang Wang

**Affiliations:** Department of Radiology, Shanxi Province People's Hospital, Taiyuan, Shanxi Province, China.

**Keywords:** breast cancer, fertility factors, mammographic density, quantra

## Abstract

To determine the association between mammographic density (MD) and the risk of breast cancer (BC) in Chinese women and to investigate the role of fertility risk factors in regulating the relationship between MD and BC.

We used Quantra software and the BI-RADS classification to assess MD in 466 patients and 932 controls. Conditional matched logistic multiple regression analysis was used to determine the relationship between MD and BC, and risk was evaluated with the odds ratio (OR) and 95% confidence interval (CI).

The ORs for category 4 versus category 2 were 1.95 (95% confidence interval [95% CI] (1.42∼2.66)) and 1.76 (95% CI (1.28∼2.42)) for the BI-RADS and Quantra classifications, respectively. The ORs for category 5 volumetric breast density (VBD) versus category 2 VBD and 5 fibroglandular tissue volume (FGV) versus category 2 FGV were 1.63 (95% CI (1.20∼2.23)) and 1.92 (95% CI (1.40∼2.63)), respectively. Females with category 5 VBD whose age at menarche was ≤13 years had the highest risk of BC (OR = 2.16, 95% CI (1.24∼3.79)), and females with category 5 FGV whose age at menarche was = 15 years had the lowest risk of BC (OR = 1.65, 95% CI (1.05∼2.62)). Females with categories 3–5 VBD and categories 3–5 FGV had reduced risks of BC with increasing number of births. Females with category 5 VBD had an increased risk of BC with increasing age at first childbirth (the OR increased from 1.49 to 1.95). Those with category 5 VBD had a reduced risk of BC with increasing breastfeeding duration (the OR decreased from 2.08 to 1.55). Females with category 5 FGV had a reduced risk of BC with increasing breastfeeding duration (the OR decreased from 4.12 to 1.62).

Both the BI-RADS density classification and Quantra measures indicated that MD is positively associated with the risk of BC in Chinese women and that associations between MD and BC risk differ by age at menarche, parity, age at first childbirth and breastfeeding duration.

## 1 Introduction

As the most common malignant tumor, breast cancer (BC) has a mortality rate second only to that of lung cancer and seriously affects the health of women. According to global cancer statistics released by GLOBCAN in 2018, it was estimated that there would be 209 million new cases of and 630,000 deaths from BC worldwide in 2018, accounting for 24.2% and 15.0% of all new cancer cases and deaths, respectively.^[1]^ Based on 72 cancer data centers in China, Chen et al predicted that in 2015, there would be 268,600 new cases of and 69,500 deaths from BC, accounting for 15.1% and 6.9% of all new cancer cases and deaths, respectively.^[2]^ Currently, BC is the cancer with the highest incidence among Chinese females (between 30 and 59 years old) and is the sixth most common cause of cancer death (the second among females <45 years of age).^[2]^ Compared to the incidences of BC among European and American females, the incidence of BC in Chinese females is low, but the rate of increase in the incidence is more than 2 times that of Caucasian females and is especially prominent in urban areas.^[3]^ The reasons may be due to the unique birth policy in China, the delayed birth of the first child, a reduced breastfeeding time and a Western lifestyle. However, compared with women in Europe and the United States, women in China are usually diagnosed with late-stage BC, and thus, the mortality rate is high.

The BI-RADS classification is a well-accepted method used by radiologists to assess mammographic density (MD). The studies on MD and BC risk showed that compared to that in females with low breast density (<5%), the risk of BC in females with extremely dense breast tissue (>75%) is increased by approximately 3–5 times.^[4,5]^ However, studies of the risk association between dense breast tissue and BC have mainly been conducted with Caucasian females.^[6–9]^ The incidence of BC among Chinese females is relatively low, and as a result, less attention has been given to MD in this population. However, in recent years, the incidence of BC in Chinese women has been increasing yearly,^[10,11]^ and the ratio of individuals with dense breast tissue is higher among Chinese women than among Caucasian women.

The accuracy of the MD measurement affects the relationship between MD and BC. Previously, the most widely used methods were the BI-RADS classification and computer-assisted area measurement using Cumulus software; however, both techniques produce variable results and lack inter- and intra-evaluator reliability, which affects the accuracy of the risk assessment between the 2 factors.^[12–14]^ As full-field digital mammography (FFDM) is currently the primary means of BC screening in women, fully automated volume density measurement software such as Volpara, developed in the Netherlands, and Quantra, developed by Hologic, Inc., in the United States, have proven to be effective MD measurement tools, and most studies have shown that there is good correlation and agreement between the fully automated volume density measurement software and the BI-RADS classification.^[15,16]^ In recent years, several studies have analyzed the risk association between MD and BC using fully automated density software.^[17–19]^ However, the relationship between MD and BC in Chinese women has not been established. Therefore, the objective of this study was to evaluate the risk relationship between MD and BC in women in Shanxi, China, using Quantra volume density software and the 5th edition of the BI-RADS density classification.

## 2 Materials and methods

### 2.1 Data collection

This study included a retrospective case-control study nested with large breast screening programs at Shanxi Provincial People's Hospital, which is the largest center for breast disease diagnosis and treatment in the area. From March 2013 to May 2017, 45,369 Han Chinese women underwent an FFDM examination at Shanxi Provincial People's Hospital, which served as the underlying cohort. Incident BCs (n = 466) were eligible during the study period. All patients were diagnosed with BC by surgical pathology or biopsy.

The control group derived from healthy females who had a breast screening during the same period of time. Inclusion criteria included:(a) a negative mammographic and ultrasound report (BI-RADS 1-2 category);(b) a negative mammographic and ultrasound report 2 years later. Exclusion criteria included: (a) a history of BC ever;(b) BI-RADS 3 category or greater in mammography or ultrasound reports;(c) a history of breast implants or surgery. A total of 932 individuals (ratio of 1:2, case:control) were randomly selected from the 1901 eligible individuals based on age (±3 years) and the FFDM examination date. The age range of the case group was 26∼81 years, and the median age was 50.86 years. The age range of the control group was 26∼81 years, and the median age was 50.06 years.

Questionnaires were used to obtain general information, including height, body weight, educational level, occupation, marital status and birthplace; fertility factors, including age at menarche, age at first birth, menstrual status, age at menopause, history of breastfeeding and history of oral contraceptives; and other factors, including history of oral estrogen, history of smoking, history of drinking and family history of BC in immediate family members. For variables with missing data (i.e., age at menarche), we plugged in data using the average value for the variable among controls in the same age group. This study was approved by the Ethics Committee of Shanxi Provincial People's Hospital, and all individuals participated in the study voluntarily. All participants were required to sign an informed consent form and to complete research-related questionnaires.

### 2.2 Breast density measures

The Hologic Selenia Mammography System and Quantra (version 2.0; Hologic, Bedford, MA) volumetric density measurement software were used. Each participant underwent examination of both breasts at the standard cranial-caudal and mediolateral-oblique positions. The images of all participants were first evaluated independently by 2 radiologists with more than 10 years of experience, using a double-blind method for the BI-RADS density classification according to the fifth edition of the BI-RADS classification standard. A third senior physician assessed the images of participants whose classifications were not consistent between the 2 radiologists so that a consensus classification was reached among the 3 physicians. The raw imaging data of all participants who underwent mammography were transferred to the breast workstation and the density software processing platform (Cenova DICOM server) for raw data processing. Finally, the Quantra density software automatically generated the bilateral breast Fibroglandular tissue volume (FGV), breast volume and volumetric breast density (VBD) percentage and provided a BI-RADS-like category of the volume density.

### 2.3 Statistical analysis

In the analysis of the general characteristics of all 1398 subjects in the case-control groups, the paired t-test was used for continuous variables, and the *χ*2 test was used for categorical variables. In the analysis of the association between the quintile classification of breast FGV, the quintile classification of the breast VBD percentage among the 1901 normal subjects and the risk factors for BC, continuous variables were analyzed for linear trends using 1-way ANOVA, and categorical variables were analyzed for linear trends using the *χ*2 test.A conditional logistic regression model was used to analyze the BI-RADS density classification, Quantra density classification, breast FGV quintile classification, VBD percentage quintile classification and the risk of BC between the case and control groups. Finally, using a conditional logistic regression model, we analyzed the risk association of the FGV quintile classification and VBD percentage quintile classification with BC after stratification by fertility factors in the case and control groups. The age at menarche (≤13, 14, or ≥15 years), number of births (1, 2, or ≥3), age at first childbirth (≤25 years or >26 years), breastfeeding time (1–12 months or >12 months), OR value and 95% confidence interval (CI) were used to describe the risk relationship. Values were considered statistically significant when *P* was < .05. All statistical analyses were carried out using SPSS version 22.0 software.

## 3 Results

As shown in Table 1, there was no significant difference in age, age at menarche, age at first childbirth or number of births between the case and control groups. Compared to the control group, the case group had a higher body mass index (BMI) and a statistically significantly higher ratio of postmenopausal patients (63.9% vs 52.0%). The BI-RADS classification and Quantra classification of the MD between the case and control groups were significantly different, and both evaluation methods showed that the ratios of density classifications 3∼4 in the case group were significantly higher than those in the control group. The average FGV and VBD percentage for the case group were both higher than those of the control group, with significant differences between the groups.

**Table 1:** General characteristics of the case group and control group.

Characteristics	Case group (466 cases)	Control group (932 cases)	*P* value
Age	50.22 (26∼81)	50.06 (26∼81)	.252
BMI	24.18	23.95	.000
Age at menarche	14.57	14.51	.126
Age at first childbirth	24.83	24.85	.149
Menstrual status			.000
Premenopausal	298 (63.9)	485 (52.0)	
Postmenopausal	168 (36.1)	447 (48.0)	
Number of births			.380
0	14 (3.0)	28 (3.0)	
1	187 (40.1)	417 (44.7)	
2	188 (40.3)	336 (36.1)	
≥3	77 (16.5)	151 (16.2)	
BI-RADS classification			.000
a. completely fat	34 (7.3)	137 (14.7)	
b. scattered dense areas	94 (20.2)	262 (28.1)	
c. heterogeneously dense	228 (48.9)	411 (44.1)	
d. extremely dense	110 (23.6)	122 (13.1)	
Quantra classification			.000
1. completely fat	13 (2.8)	48 (5.2)	
2. scattered dense areas	124 (26.6)	321 (34.4)	
3. heterogeneously dense	247 (53.0)	482 (51.7)	
4. extremely dense	82 (17.6)	81 (8.7)	
Quantra VBD percentage (median)	18.41 (4.00∼66.90)	15.97 (2.00∼60.00)	.020
Quantra dense volume (median)	117.63 (16.50∼502.50)	95.73 (8.50∼333.00)	.000

As shown in Table 2, the relationship between the quintile classification for breast FGV or VBD percentage and the risk factors for BC was determined. Age, BMI and fertility factors had linear associations with breast FGV and VBD percentage, while oral contraceptives and family history did not. Younger females had a greater FGV and higher VBD percentage. Females with a higher BMI had a lower VBD percentage but a higher FGV. Compared to postmenopausal women, premenopausal women had a greater FGV and higher VBD percentage. Women who were younger at menarche and who were older at first childbirth had a greater FGV and higher VBD percentage. Compared to women who had previously given birth, women who had not given birth had a greater FGV and higher VBD percentage. Women who had more births (≥ 3) had a lower VBD percentage and lower FGV. Among women who had previously given birth, women who did not breastfeed had a higher VBD percentage and greater FGV. Among women who did breastfeed, women who breastfed ≤ 12 months had a higher VBD percentage and greater FGV, and women who breastfed > 12 months had a lower VBD percentage and less FGV.

**Table 2:** Relationship between VBD percentage or FGV and the risk factors for breast cancer.

	VBD percentage (%)						FGV (cm^3^)					
	Q1 (<10) N = 379	Q2 (10–14.0) N = 379	Q3 (14.1–18.5) N = 381	Q4 (18.6–23.6) N = 379	Q5 (≥23.7) N = 383	*P*_trend_	Q1 (<57) N = 360	Q2 (57–78) N = 393	Q3 (79–103) N = 376	Q4 (104–135) N = 386	Q5 (≥136) N = 386	*P*_trend_
Age (yr, mean)	54.5	50.1	46.0	45.1	43.5	0.000	51.9	49.2	47.1	45.6	45.7	.000
BMI (kg/m^2^,mean)	24.78	24.49	23.50	23.08	22.42	0.000	23.40	23.56	23.75	23.72	23.81	.042
Menarche (yr, mean)	14.78	14.28	14.43	14.39	14.29	0.002	14.71	14.45	14.35	14.39	14.29	.002
Age at first birth (yr, mean)	24.58	24.96	24.83	25.15	25.30	0.003	24.70	24.66	25.14	24.98	25.34	.004
Number of births (%)												
0	15	20.0	20.0	15	30	0.000	11.7	11.7	23.3	31.7	21.7	.000
1	14.2	20.3	21.2	20.6	23.8		16.3	21.3	19.5	20.3	22.7	
2	21.2	19.0	19.6	21.1	19.1		19.9	19.7	20.8	19.9	19.7	
≥3	37.8	21.2	15.4	15.4	8.1		27.4	23.2	17.4	18.9	13.1	
Breastfeeding (mo, %)												
0	22.0	16.7	16.1	21.4	23.8	0.000	17.9	18.5	17.9	20.2	25.6	.015
1–12	12.9	19.5	22.0	22.2	23.4		15.6	21.8	21.7	20.2	20.7	
>12	23.6	20.9	19.6	18.6	17.3		21.3	20.8	18.9	19.9	19.2	
Menstrual status (%)												
Premenopausal	8.2	15.1	23.7	25.6	27.4	0.000	12.5	18.8	20.6	23.8	24.3	.000
Postmenopausal	37.9	27.3	14.5	11.2	9.2		28.7	23.5	18.5	15.0	14.2	
Oral contraceptives (%)	21.6	18.9	20.2	17.9	21.4	0.756	17.0	23.4	20.9	20.2	18.4	.633
Family history (%)	24.0	16.0	23.0	24.0	13.0	0.294	25.0	15.0	19.0	19.0	22.0	.75

As shown in Table 3, the risk relationship between MD as assessed by BI-RADS and Quantra software and BC was assessed in the case-control groups after controlling for BMI, menstrual status, age at menarche, age at first childbirth and number of births. Compared to that in females with a BI-RADS category 2 density, the risk of BC in females with category 3 density was OR = 1.37, 95% confidence interval (95% CI) (1.06∼1.77), and the risk in females with category 4 density was OR = 1.95, 95% CI (1.42∼2.66). Compared to females identified as being in Quantra category 2, females identified as being in Quantra category 3 did not have a risk of BC (OR = 1.14, 95% CI (0.90∼1.45)), and the risk of BC in females identified as being in category 4 was OR = 1.76, 95% CI (1.28∼2.42). The risks of BC in females in Quantra categories 3 and 4 were both lower than those in females in BI-RADS categories 3 and 4. The *P* values for the within-group trend analysis for both classification methods were <.0001.

**Table 3:** The association between breast density determined by BI-RADS or Quantra classification and the risk of breast cancer.

Density classification	Case/control	BI-RADS	Case /control	Quantra
a. completely fat	34/137	0.75 (0.50∼1.11)	13/58	0.66 (0.37∼1.17)
b. scattered dense areas	94/262	1.00	124/312	1.00
c. heterogeneously dense	228/411	1.37 (1.06∼1.77)	245/481	1.14 (0.90∼1.45)
d. extremely dense	110/122	1.95 (1.42∼2.66)	84/81	1.76 (1.28∼2.42)
*P* value for trend		<.0001		<.0001

As shown in Table 4, the risk relationship between VBD percentage and FGV quintile classification for the case-control groups and the risk of BC were assessed after controlling for BMI, menstrual status, age at menarche, age at first childbirth and number of births. Compared to that in females with a category 2 VBD percentage, the risk of BC in females with a category 5 VBD percentage was OR = 1.63, 95% CI (1.20∼2.23). Compared to that in females with category 2 FGV, the risk of BC in females with categories 4 and 5 FGV were OR = 1.62, 95% CI (1.18∼2.23) and OR = 1.92, 95% CI (1.40∼2.63). The *P* values for the within-group trend analysis for both VBD percentage and FGV classifications were <.0001.

**Table 4:** The association between MD in the VBD or FGV classifications and risk of breast cancer.

VBD percentage classification	Case/control	OR (95% CI)	Classification of FGV	Case/control	OR (95% CI)
1. ≤9.52	77/202	0.92 (0.67–1.28)	1. <56	63/213	1.20 (0.85–1.69)
2. 9.53–13.24	72/206	1.00	2. 56-78	74/200	1.00
3. 13.27–17.89	91/190	1.11 (0.81–1.52)	3. 79-104	90/193	1.39 (1.00–1.93)
4. 17.92-23	99/178	1.22 (0.88–1.67)	4. 104.5-141	108/174	1.62 (1.18–2.23)
5. >23	127/156	1.63 (1.20–2.23)	5. >141	131/152	1.92 (1.40–2.63)
*P* value for trend		<.0001			<.0001

As shown in Table 5, the risk association of the FGV quintile classification and VBD percentage quintile classification with BC was determined after stratification of the fertility factors for the case and control groups, using category 2 as the reference. In the determination of the menarche risk stratification, the risk of BC in females with an age at menarche ≤13 years increased with the increase in VBD percentage category (*P* = .007), and only females with category 5 VBD (age of menarche ≤13 years) had a significantly increased risk of BC (OR = 2.16, 95% CI (1.24∼3.79)). Females in all menarche groups had an increased risk of BC with increasing FGV classification (all p≤0.024), and only those with an age at menarche ≤13 years with category 4 FGV had a significantly increased risk of BC (OR = 2.17, 95% CI (1.22∼3.88)); females with an age at menarche ≥15 years with category 5 FGV had the lowest risk of BC (OR = 1.65, 95% CI (1.05∼2.62)).

**Table 5:** The association between FGV or VBD percentage distribution after stratification of the fertility factors and the risk of breast cancer.

	VBD percentage classification											
	Q1 (≤9.52)		Q2 (9.53–13.24) Reference		Q3 (13.27–17.89)		Q4 (17.92–23)		Q5 (>23)			
Risk factors for breast cancer	NCase/control	OR (95% CI)	NCase/control	OR	NCase/control	OR (95% CI)	NCase/control	OR (95% CI)	NCase/control	OR (95% CI)	P value for trend	P value for differences between groups
Age of menarche												0.001
≤13	15/44	1.25 (0.63–2.49)	19/76	1.00	33/57	1.59 (0.89–2.83)	32/58	1.53 (0.85–2.76)	53/58	2.16 (1.24–3.79)	0.007	
14	15/51	0.87 (0.43–1.78)	18/46	1.00	11/54	0.56 (0.26–1.23)	22/37	1.30 (0.66–2.58)	31/45	1.49 (0.78–2.84)	0.079	
≥15	42/111	0.83 (0.53–1.28)	40/80	1.00	47/79	1.16 (0.75–1.80)	45/83	1.05 (0.66–1.66)	43/53	1.37 (0.85–2.21)	0.190	
Number of births												0.111
1	15/53	1.27 (0.65–2.48)	21/98	1.00	45/95	1.82 (1.08–3.09)	37/85	1.84 (1.05–3.20)	69/86	2.74 (1.64–4.60)	0.000	
2	26/84	0.65 (0.39–1.08)	36/58	1.00	34/68	0.84 (0.52–1.36)	45/70	0.95 (0.60–1.51)	47/56	1.14 (0.72–1.81)	0.288	
≥3	31/63	0.99 (0.56–1.75)	19/38	1.00	9/25	0.74 (0.32–1.68)	13/19	1.07 (0.50–2.31)	5/6	1.02 (0.35–2.96)	0.981	
Age of first childbirth												0.001
≤25	44/131	0.80 (0.53–1.19)	52/111	1.00	52/126	0.88 (0.59–1.30)	56/108	1.04 (0.70–1.55)	71/74	1.49 (1.01–2.19)	0.025	
>26	28/69	1.21 (0.69–2.12)	24/83	1.00	36/62	1.63 (0.96–2.77)	39/66	1.62 (0.95–2.77)	50/74	1.95 (1.15–3.32)	0.012	
Breastfeeding (months)												0.000
1–12	9/38	0.80 (0.35–1.82)	19/60	1.00	26/60	1.23 (0.68–2.24)	28/57	1.39 (0.76–2.53)	52/61	2.08 (1.20–3.61)	0.003	
>12	58/142	1.01 (0.69–1.48)	51/127	1.00	59/114	1.15 (0.78–1.69)	61/94	1.27 (0.85–1.89)	61/71	1.55 (1.03–2.33)	0.028	

	FGV classification											
	Q1 (≤55)		Q2 (56–78) Reference		Q3 (79–104)		Q4 (104.5–141)		Q5 (>141)			
Risk factors for breast cancer	N		N			OR (95% CI)	N	OR (95% CI)	N	OR (95% CI)	*P* value for trend	*P* value for differences between groups
	Case	OR (95% CI)	Case	OR	N		Case		Case/			
	/control		/control		Case/		/control		control			
					control							
Age of menarche												.000
≤13	12/44	1.17 (0.55–2.48)	16/68	1.00	29/63	1.57 (0.84–2.92)	47/59	2.17 (1.22–3.88)	48/59	2.15 (1.21–3.83)	.002	
14	18/50	1.53 (0.72–3.26)	12/55	1.00	24/51	1.96 (0.95–4.04)	12/41	1.30 (0.56–2.99)	31/36	2.56 (1.24–5.28)	.024	
≥15	44/106	1.03 (0.66–1.62)	35/90	1.00	37/79	1.12 (0.70–1.80)	49/74	1.38 (0.89–2.15)	52/57	1.65 (1.05–2.62)	.014	
Number of births												.000
1	12/68	0.94 (0.46–1.93)	20/102	1.00	40/84	1.93 (1.12–3.31)	51/81	2.26 (1.34–3.81)	64/82	2.58 (1.55–4.30)	.000	
2	33/78	1.11 (0.66–1.87)	25/67	1.00	35/73	1.15 (0.68–1.92)	38/64	1.27 (0.76–2.12)	57/54	1.68 (1.04–2.74)	.024	
≥3	29/50	1.30 (0.71–2.37)	17/41	1.00	12/27	1.20 (0.56–2.58)	14/21	1.37 (0.66–2.85)	5/12	0.87 (0.30–2.52)	.789	
Age of first childbirth												
≤25	50/128	1.33 (0.87–2.02)	39/142	1.00	51/109	1.48 (0.97–2.25)	65/96	1.79 (1.20–2.68)	70/75	2.10 (1.40–3.17)	.000	.000
>26	24/68	0.96 (0.53–1.71)	23/68	1.00	36/75	1.25 (0.74–2.11)	38/70	1.33 (0.79–2.25)	56/73	1.61 (0.98–2.66)	.026	
Breastfeeding (months)												
1–12	15/50	1.85 (0.81–4.27)	9/63	1.00	27/59	2.62 (1.22–5.60)	39/54	3.56 (1.71–7.41)	44/50	4.12 (1.98–8.57)	.000	.041
>12	56/132	1.14 (0.77–1.68)	48/130	1.00	56/110	1.24 (0.84–1.83)	57/97	1.29 (0.87–1.91)	73/79	1.62 (1.11–2.38)	.013	

In the analysis of the number-of-births risk stratification, only those with a number of births = 1 had an increased risk of BC with increasing VBD percentage classification (*P* = .000), and no risk of BC was identified in the group with multiple births. Similarly, in the FGV classification, females with a number of births equaling 1 or 2 had increased risks of BC with increasing FGV classification (both *P*≤.024), and those in FGV categories 3–5 had a gradually reduced risk of BC with the increase in the number of births. In the risk analysis for age at first childbirth, both age groups had an increased risk of BC with increasing VBD percentage classification (both *P* ≤.025), and females with category 5 VBD had an increased risk of BC with increasing age at first childbirth (OR increased from 1.49 to 1.95). All age at first childbirth groups had an increased risk of BC with increasing FGV classification (both *P* ≤.026), and those with categories 4 and 5 FGV had an increased risk of BC with increasing age at first childbirth.

In the stratification of breastfeeding duration among those who had given birth, females in all breastfeeding duration groups had an increased risk of BC with increasing VBD percentage classification (both *P* ≤ .028), and those with category 5 VBD had a reduced risk of BC with increasing breastfeeding duration (the OR decreased from 2.08 to 1.55). Females in all breastfeeding duration groups had an increased risk of BC with increasing FGV classification (both *P* ≤ .013), and those with categories 4 and 5 FGV had reduced risks of BC with increasing breastfeeding duration.

## 4 Discussion

In the analysis of digital mammographic images of all participants in the case and control groups, we found that both MD assessment methods, namely, the 5th edition of BI-RADS and the Quantra software, showed a positive correlation between MD and BC risk, which is consistent with previous studies.^[17,18]^ Using category 2 as the reference, neither of the assessment methods found a decreased risk of BC in females in category 1, which might be related to an insufficient sample size. We found that compared to the BI-RADS density classification, the BI-RADS-like category of the Quantra software showed reduced risk values for the association between MD and BC. The OR values for BC risk in females in category 4 with BI-RADS and Quantra were 1.95 and 1.76, respectively. For females in category 3 for both methods, the BI-RADS classification predicted that the OR of the BC risk was 1.37, and the Quantra method did not show any significant risk association.

A case-control study by Brandt et al. assessed the association between the BI-RADS-like category of the Quantra software, the quintile classifications of VBD percentage and FGV, and the risk of BC.^[19]^ Compared to females identified as having Quantra density category 2, females identified as having categories 3 and 4 had increased risks of BC (OR values of 1.40 and 1.94, respectively). Compared to females with category 2 VBD percentages, females in categories 4 and 5 had increased risks of BC (OR values of 1.66 and 1.78, respectively). Similarly, compared to females identified as being in FGV category 2, females in category 5 had an increased risk of BC (OR = 1.52).^[19]^ For the BI-RADS-like category of the Quantra software, our results showed decreased risk values for the association between MD and BC compared with the results from the study by Brandt. We further assessed the risk association between the VBD percentage, the FGV quintile classification and the risk of BC in the case and control groups. We found that compared to females in VBD percentage category 2, females in category 5 had an increased risk of BC (OR = 1.63) and that compared to females with category 2 FGV, females identified as having categories 4 and 5 FGV had increased risks of BC (OR = 1.62 and 1.92, respectively). These findings were consistent with those in the study by Brandt et al. A case-control study by Eng et al. also assessed the correlation between the VBD quintile classification using Quantra software and the risk of BC^[20]^ and showed that compared with females in category 1, females in categories 4 and 5 had increased risks of BC (OR = 3.43 and 3.94, respectively), while there was no significant association between FGV quintile classification and the risk of BC.

Compared to that of Caucasian females, the percentage of Chinese females with BI-RADS category 3-4 density, which is especially prominent among premenopausal females, is higher.^[21–23]^ Our study showed that the proportion of females in category 3, as determined by both density assessment methods, was highest. The conventional BI-RADS density classification method relies on the subjective evaluation of radiologists, which produces variable results and lacks inter- and intra-evaluator reliability.^[15,24]^ Second, this type of evaluation is affected by image characteristics because the images to be evaluated are generated after reconstruction of the raw data. The majority of studies show moderate or good agreement in the weighted Kappa analysis between the 5th edition of the BI-RADS classification and the Quantra software.^[25–28]^ Compared to the BI-RADS classification, the MD classification acquired through analysis of the raw data using Quantra volumetric density measurement software has better stability and repeatability.^[29]^ A study by Wang et al. showed that the Quantra density measurements had a good correlation with MRI breast density measurements and could realistically reflect the mammographic dense tissue volume.^[30]^ Therefore, Quantra volumetric density measurement software can more accurately reflect the risk association between MD and BC.

Earlier studies suggested that there was a risk relationship between fertility factors and BC. More births and earlier age at first childbirth could reduce the risk of hormone receptor-positive BC in females,^[31,32]^ and breastfeeding for longer times could reduce the risks of hormone receptor-positive and triple-negative BC.^[33]^ Some studies used the semiautomated area measurement method in Cumulus software to assess the relationship between female fertility factors and MD. The studies showed that the number of births and age at first childbirth were negatively correlated with MD^[34]^ and that breastfeeding time was positively associated with the percentage of dense fibrous breast tissue and dense fibrous area.^[35,36]^ However, the mechanism of the influence of fertility factors on MD is not yet clear. Two previous studies in a large-scale survey from Chinese females of Han ethnicity analyzed the risk association between fertility factors among the healthy population and MD using BI-RADS density classification and found that the relationship between fertility factors and MD distribution was consistent with the distribution relationship for Caucasian females.^[21,22]^ Our study investigated the relationship between VBD percentage and FGV quintile classification and the risk factors for BC among 1901 healthy individuals. We found that age and menstrual status had a linear negative association with breast VBD percentage classification and FGV classification and that BMI had a linear negative association with breast VBD percentage classification and a linear positive association with breast FGV classification, which was consistent with a previous study.^[17]^ We also found that menarche and number of births had a linear negative association with VBD percentage classification and FGV classification and that age at first childbirth had a linear positive association with VBD percentage classification and FGV classification, and these findings were consistent with those of previous studies.^[34,35]^ Finally, we found that breastfeeding time had a linear negative association with VBD percentage classification and a weak linear negative association with FGV classification, which was not consistent with the previous study.^[35]^ Therefore, further studies are required to determine the relationship between breastfeeding time and VBD percentage and FGV classifications.

Fertility factors not only are associated with the risk of BC but also affect only the VBD percentage and FGV distribution of MD among the healthy population, and a higher MD is positively associated with the risk of BC. It is not clear whether fertility factors regulate the risk relationship between MD and BC. Two previous case-control studies assessed the relationships among fertility factors, MD and BC. The study by Woolcott et al. found that among females with high MD, those who had given birth had a lower risk of BC than those who had never given birth.^[36]^ A study by Yaghjyan showed that among females with high MD, compared to those who had given birth to 1 child, those who had multiple births (2 or ≥3) had a lower risk of BC.^[37]^ Our results also showed that among females with a high VBD percentage and high FGV classification, compared to those who had given birth to 1 child, females who had given birth to multiple children had a lower risk of BC, which is consistent with previous studies. Our results showed that with a delay in age at menarche, females with a high VBD percentage and high FGV classifications had a lower risk of BC and that with an increase in age at first childbirth, females with a high VBD percentage had an increased risk of BC; however, females with high FGV classifications had a lower risk of BC, which is consistent with a previous study.^[37]^ Among the population of women who had breastfed previously, compared to females with short durations of breastfeeding (1–11 months), longer breastfeeding durations (≥12 months) resulted in a reduced risk of BC in females with high VBD percentage classifications and high FGV classifications.

Our study analyzed the association between MD and the risk of BC using the BI-RADS classification and Quantra software to assess MD in case and control groups and confirmed that females of Han ethnicity in Shanxi, China with high MD had an increased risk of BC. The relationship shown in this study between MD and the risk factors for BC among the healthy population is consistent with that found in previous studies, which further confirms the correlation between the distribution of MD among healthy females of Han ethnicity and fertility factors. Finally, we further investigated whether fertility factors could regulate the risk relationship between MD and BC and confirmed this regulation by fertility factors, which could provide a basis for the prevention and treatment of BC.

This study has some limitations. We used only Quantra software to assess MD; other software that measures MD should be used to further confirm the association between MD and BC risk in women of Han ethnicity. Moreover, the reference population for establishing the Quantra BI-RADS-like category mainly comprised Caucasian women, and the exact cut-off point thresholds are not specified. Indeed, the use of Quantra density software is still in the preliminary stage. Additionally, this case-control study had a limited sample size; a study with a larger sample size is needed to further confirm our findings.

In summary, by assessing the MD of Han Chinese women in Shanxi, China, using the BI-RADS and Quantra methods in a case-control study, we found that women with higher MD classifications had an increased risk of BC and that fertility factors regulated this risk relationship. Further studies are required to confirm this regulation.

## Acknowledgments

This study was supported by the Department of Breast Surgery and Pathology at Shanxi Provincial People's Hospital.

## Author contributions

H.G. Zhao designed the experiments; F.Y. Wang and T.T. Liu administered all questionnaires; and J.M. Wang analyzed all data and wrote the manuscript.

**Conceptualization:** Hong Guang Zhao.

**Data curation:** Tong Tong Liu, Fei Yang Wang.

**Funding acquisition:** Jian Ming Wang.

**Project administration:** Jian Ming Wang.

**Writing – original draft:** Jian Ming Wang.

**Writing – review & editing:** Jian Ming Wang.
